# Short-Term Clinical Outcomes of Single Versus Dual Antiplatelet Therapy after Infrainguinal Endovascular Treatment for Peripheral Arterial Disease

**DOI:** 10.3390/jcm9113515

**Published:** 2020-10-30

**Authors:** Jetty Ipema, Rutger H. A. Welling, Olaf J. Bakker, Reinoud P. H. Bokkers, Jean-Paul P. M. de Vries, Çagdas Ünlü

**Affiliations:** 1 Department of Surgery, Northwest Clinics, Wilhelminalaan 12, 1815 JD Alkmaar, The Netherlands; cagdas.unlu@nwz.nl; 2 Department of Surgery, St. Antonius Hospital, Koekoekslaan 1, 3435 CM Nieuwegein, The Netherlands; r.welling@antoniusziekenhuis.nl (R.H.A.W.); o.bakker@antoniusziekenhuis.nl (O.J.B.); 3 Department of Radiology, Medical Imaging Center, University Medical Center Groningen, University of Groningen, Hanzeplein 1, 9713 GZ Groningen, The Netherlands; r.p.h.bokkers@umcg.nl; 4 Department of Surgery, Division of Vascular Surgery, University Medical Center Groningen, University of Groningen, Hanzeplein 1, 9713 GZ Groningen, The Netherlands; j.p.p.m.de.vries@umcg.nl

**Keywords:** peripheral arterial disease, antiplatelet therapy, endovascular intervention, acetylsalicylic acid, clopidogrel

## Abstract

After infrainguinal endovascular treatment for peripheral arterial disease (PAD), it is uncertain whether single antiplatelet therapy (SAPT) or dual antiplatelet therapy (DAPT) should be preferred. This study investigated major adverse limb events (MALE) and major adverse cardiovascular events (MACE) between patients receiving SAPT and DAPT. Patient data from three centers in the Netherlands were retrospectively collected and analyzed. All patients treated for PAD by endovascular revascularization of the superficial femoral, popliteal, or below-the-knee (BTK) arteries and who were prescribed acetylsalicylic acid or clopidogrel, were included. End points were 1-, 3-, and 12-month MALE and MACE, and bleeding complications. In total, 237 patients (258 limbs treated) were included, with 149 patients receiving SAPT (63%) and 88 DAPT (37%). No significant differences were found after univariate and multivariate analyses between SAPT and DAPT on 1-, 3-, and 12-month MALE and MACE, or bleeding outcomes. Subgroup analyses of patients with BTK treatment showed a significantly lower 12-month MALE rate when treated with DAPT (hazard ratio 0.33; 95% confidence interval 0.12–0.95; *p* = 0.04). In conclusion, although patient numbers were small, no differences were found between SAPT and DAPT regarding MALE, MACE, or bleeding complications. DAPT should, however, be considered over SAPT for the subgroup of patients with below-the-knee endovascular treatment.

## 1. Introduction

According to the latest Global Burden of Disease Study, peripheral arterial disease (PAD) ranks second as the most prevalent cardiovascular disease in the world [1]. Treatment is based on lifestyle changes, optimization of medication, and revascularization. Currently, endovascular treatment is preferred over surgery for patients with symptomatic PAD because of its less invasive nature and lower morbidity rates [2,3]. In these patients with high cardiovascular risk factors, antiplatelet therapy is prescribed for reducing cardiovascular events [4,5]. After an endovascular intervention, additional antiplatelet therapy is also indicated for preventing thromboembolic complications, thus improving patency and limb salvage [6].

Antiplatelet agents can be prescribed as single antiplatelet therapy (SAPT) or dual antiplatelet therapy (DAPT). Different surveys among interventionalists worldwide have, however, shown that there is wide variety in prescription patterns [7,8,9,10]. Whereas most preferred DAPT over SAPT, especially after stenting, the current Global Vascular Guideline, a 2019 international guideline by the European Society for Vascular Surgery, the Society for Vascular Surgery, and the World Federation of Vascular Societies, does not provide a specific recommendation for DAPT after infrainguinal stenting [11]. Besides differences between therapists, guideline recommendations show discrepancies on indication and duration of DAPT, and specific recommendations are based on low-level evidence [2,3,11,12].

The inconsistencies between antiplatelet prescription and current guidelines reflect a lack of compelling evidence in the literature. To date, there is no clear-cut answer whether DAPT should be preferred over SAPT after endovascular treatment of the superficial femoral artery (SFA), with or without (drug-eluting) stents [13]. This paucity of data is also recognized for treatment of below-the-knee (BTK) arteries, with a preference in current practice for DAPT in the more distally located lesions [7,8,9,10]. 

Therefore, the aim of this retrospective, multicenter study was to investigate 1-, 3-, and 12-month major adverse limb events (MALE) and major adverse cardiovascular events (MACE) between SAPT and DAPT after endovascular revascularizations of the femoropopliteal and BTK tract.

## 2. Experimental Section

### 2.1. Study Design

This was a retrospective study performed at three centers in the Netherlands: The Northwest Clinics Alkmaar, the St. Antonius Hospital Nieuwegein, and the University Medical Center Groningen. The Medical research Ethics Committees United (W19.106) and the local research department of each hospital approved the study. Study data were obtained from electronic patient records.

### 2.2. Study Population

Consecutive patients who had undergone endovascular interventions of the lower limbs between 1st January 2017 and 1st January 2018 at the Northwest Clinics Alkmaar or the St. Antonius Hospital Nieuwegein, or between 1st January 2015 and 1st April 2018, at the University Medical Center Groningen were analyzed. To include the same number of patients from each hospital, the study period of the last center was extended. Patients were included when treated for lesions located in the SFA, popliteal artery, or BTK arteries (including popliteal artery segment 3, tibioperoneal trunk, anterior tibial artery, posterior tibial artery, and peroneal artery) to treat PAD and who were using acetylsalicylic acid or clopidogrel, or a combination of acetylsalicylic acid with clopidogrel after the intervention. All Rutherford categories (1–6) were included. Patients with an acute worsening of chronic PAD, unsuccessful recanalizations, endovascular intervention for reason other than PAD, antithrombotic therapy other than acetylsalicylic acid or clopidogrel, and loss to follow-up <30 days were excluded. Patients were included in the SAPT group when prescribed acetylsalicylic acid 80 mg daily or clopidogrel 75 mg daily, and were included in the DAPT group when prescribed acetylsalicylic acid 80 mg daily and clopidogrel 75 mg daily.

### 2.3. End Points

Primary end points were 1-, 3-, and 12-month MALE. Secondary end points were 1-, 3-, and 12-month MACE, and bleeding complications. Definitions of the end points are shown in Table 1.

Subgroup analyses were performed in patients with femoropopliteal treatment to test for stent use, stent type, and stent length. Subgroup analysis was also performed for patients with BTK treatment.

### 2.4. Statistical Analysis

Baseline characteristics and procedural data in patients treated with SAPT were compared with those in patients treated with DAPT. Continuous variables are expressed as the mean with the standard deviation if normally distributed, and as the median with the range if non-normally distributed. Normally distributed data were compared using the independent samples *t* test and non-normally distributed data using the Mann–Whitney *U* test, as appropriate. Categorical variables are expressed as number (%) and were compared using the χ^2^ or Fisher exact test, as appropriate. Cox regression analysis was performed to calculate differences in MALE and MACE between SAPT and DAPT groups. In case patients were treated more than once during the follow-up, only the first procedure on the limb was used for MALE and the first procedure within the patient for MACE. For both MALE and MACE, follow-up ended early in case of unforeseen changes in antiplatelet therapy, or for patients in the DAPT group, when DAPT was converted to SAPT according to prescription.

Multivariate logistic regression was performed to adjust for confounders. Known risk factors for MALE and MACE (including age, sex, tobacco use, diabetes mellitus, hyperlipidemia, hypertension, ischemic heart disease, prior stroke, renal failure, dialysis dependency, American Society of Anesthesiologists Physical Status Classification, and Rutherford category), hospital site, and baseline variables with a univariate predictive value of *p* < 0.1 were candidates for entering the multivariate regression analysis. In the subgroup of patients with femoropopliteal treatment, stent use, stent type, and stent length were also candidates for entering the multivariate regression analysis. Results are shown as the hazard ratio (HR) with the 95% confidence interval (CI). Bleeding complications between SAPT and DAPT were calculated with χ^2^ test. Statistical significance was defined as *p* < 0.05. Statistical analysis was performed using IBM SPSS Statistics 27 software (IBM, Armonk, NY, USA).

## 3. Results

### 3.1. Study Population 

**Figure 1:**
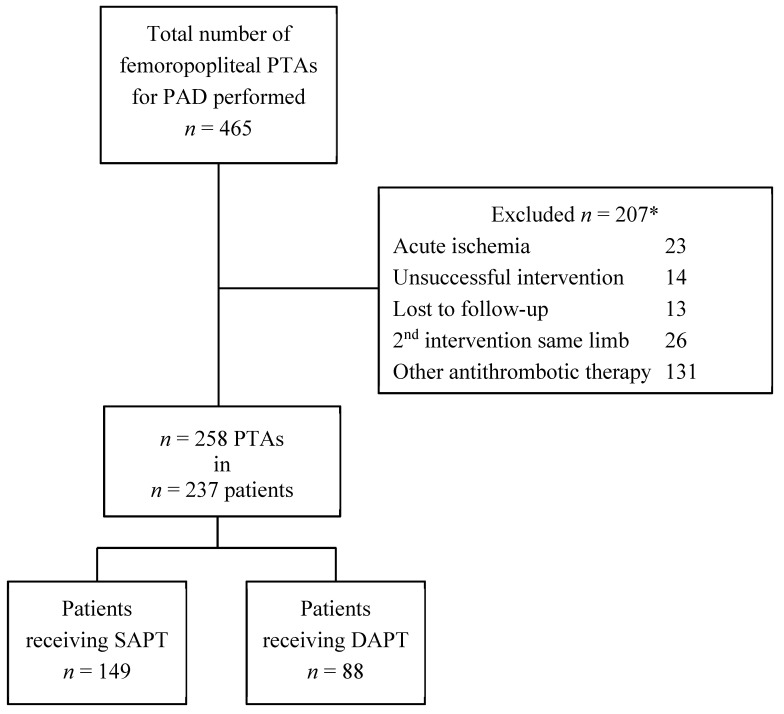
Flowchart of the inclusion process. DAPT = dual antiplatelet therapy; *n* = number; PAD = peripheral arterial disease; PTA = percutaneous transluminal angioplasty; SAPT = single antiplatelet therapy. * Patients could have more than one reason to be excluded; only one reason was registered.

During the study period, 465 femoropopliteal endovascular interventions for PAD were performed in the three centers. After excluding interventions that did not meet the inclusion criteria, 258 interventions performed in 237 patients remained (Figure 1). Of these, 149 patients received SAPT and 88 DAPT. The DAPT group was significantly younger (68.7 ± 12.1 years vs. 74.4 ± 11.1 years) and had a higher percentage of former and current tobacco users (85% vs. 66%) and a higher proportion of patients with hyperlipidemia (69% vs. 44%). Femoropopliteal stent use was significantly higher in the DAPT than in the SAPT group (76% vs. 40%, *p* < 0.01), but stent length did not differ significantly (*p* = 0.65). After bare-metal stent (BMS) placement, more patients received SAPT than DAPT (74% vs. 46%), but after drug-eluting stent (DES) placement, more patients received DAPT than SAPT (44% vs. 21%). DAPT durations varied from at least 3 months to 12 months, and 34 patients ended DAPT <12 months according to prescription. Other baseline characteristics of patients and limbs are reported in Table 2 and Table 3. 

### 3.2. Primary end Point

**Figure 2:**
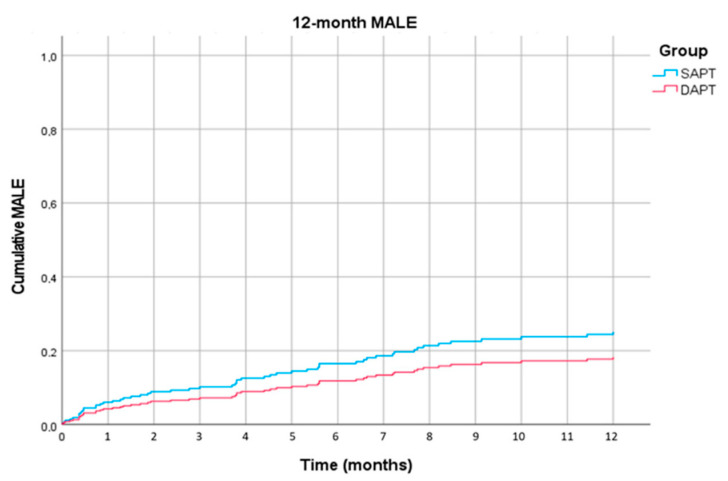
Multivariate analysis of 12-month major adverse limb event (MALE) outcomes between patients receiving single antiplatelet therapy (SAPT) and patients receiving dual antiplatelet therapy (DAPT) (HR, 0.70; 95% CI, 0.39–1.25; *p* = 0.23).

Comparing DAPT vs. SAPT, 1- and 3-month MALE did not differ significantly in univariate analysis or after adjusting for confounders. MALE at 12 months also did not differ significantly between the groups in univariate (HR, 0.72; 95% CI, 0.40–1.29; *p* = 0.27) and multivariate analysis (HR, 0.70; 95% CI, 0.39–1.25; *p* = 0.23) adjusted for diabetes mellitus, renal failure, and the presence of chronic limb-threatening ischemia (Figure 2).

Subgroup analyses of patients who underwent BTK treatment showed no significant differences in 1- and 3-month MALE. The 12-month univariate analyses of MALE in this subgroup was also not significantly different, but when corrected for age, 12-month MALE was lower for the DAPT group than for the SAPT group (HR, 0.33; 95% CI, 0.12–0.95; *p* = 0.04).

The 1-, 3-, and 12-month results did not differ significantly between SAPT and DAPT for subgroup analyses of patients who had femoropopliteal and derivatives of MALE (major amputation, target lesion revascularization (TLR), or target vessel revascularization (TVR)). All results are presented in Table 4.

### 3.3. Secondary end Points

No significant differences were found between SAPT and DAPT for 1-, 3-, and 12-month MACE or derivatives from MACE (death, myocardial infarction, or stroke) in both univariate and multivariate analyses (Table 5). 

A bleeding event occurred in three patients (2.0%) in the SAPT group, of which one was classified as BARC 2 and two as BARC 3. Four patients (4.5%) in the DAPT group had a bleeding event, of which one patient was classified as BARC 1, one as BARC 2, and two as BARC 3. Bleeding rates did not significantly differ between SAPT and DAPT (*p* = 0.43).

## 4. Discussion

This study evaluated real-world clinical outcomes between patients receiving SAPT and DAPT after infrainguinal endovascular treatment. No favorable effect of the use of DAPT compared with SAPT was shown on 1-, 3-, and 12-month MALE and MACE in the total patient cohort. DAPT was, however, found to be associated with a significantly lower 12-month MALE rate than SAPT in the subgroup of patients with BTK treatment.

The lower 12-month MALE rate with DAPT is an interesting finding that underlines the international preference of DAPT prescription over SAPT after BTK treatment, as shown by a recent survey [7]. However, the respondents from this survey also answered that the decision to choose DAPT is not evidence based and that guidelines do not provide specific recommendations. Therefore, the reason for the preference for DAPT remains unknown. The current study suggests that DAPT could indeed be favorable after BTK treatment, but even though it included the highest proportion of BTK interventions compared with previous research, subgroups were small, and conclusions should be taken with caution.

In the same survey, most of the respondents answered a preference for prescribing DAPT over SAPT after stenting, which was also observed in the current study. Prescription patterns varied between the centers included in this study. The prescription of DAPT was higher in one center, which was related to the use of DESs. In currently available literature, only one study showed a lower amputation rate for DAPT vs. SAPT in a subgroup of patients with diabetes mellitus who underwent stenting [15]. The current real-world study found no advantage of DAPT over SAPT on MALE when taking into account diabetes mellitus, the use of stents, type of stent, or stent length.

Regarding endovascular treatment of the SFA and popliteal artery, only one randomized trial has been performed that studied SAPT vs. DAPT (MIRROR trial). The trial showed fewer TLRs in the DAPT group at 6 months [16,17]. The DAPT group received DAPT for 6 months compared with at least 3 months in the current study. Although our study showed a trend toward lower TLR and TVR rates after 3 months in favor of the DAPT group, the difference was not statistically significant. Similar to the MIRROR trial, the current study found no significant difference between DAPT and SAPT in 12-month TLR rates. 

Although the 6-month mortality rate in the MIRROR trial was not significantly different between DAPT and SAPT, the 12-month mortality rate was lower in the DAPT group [16,17]. However, this trial consisted of a small study population of only 40 patients in each group. The lower mortality rate for the DAPT group was not demonstrated in our study, despite many patients receiving DAPT for 12 months in contrast to the 6 months in the MIRROR trial.

Another gap in the literature is the optimal duration of DAPT. Whereas guidelines recommend DAPT for 1 month, in randomized trials studying outcomes of different types of stents or balloons, DAPT duration varied from 2 months to 1 year [18,19]. The optimal duration of DAPT, therefore, remains a major question, and the lack of strong evidence reflects the different DAPT durations in our study. To date, studies have shown no favorable effect of longer vs. shorter DAPT duration in 1-year outcomes [20,21,22]. One study showed lower 5-year MALE and MACE rates when DAPT was prescribed for >6 months compared with <6 months or SAPT [23]. A recently published study, similar to ours, compared 3-month DAPT with SAPT and showed no significant difference in amputation-free survival [24]. Different from our study, it included a high proportion of patients treated for aorta-iliac lesions and only a few patients with stents. Currently, two ongoing clinical trials are investigating short vs. long DAPT after endovascular intervention [25,26].

Although antiplatelet therapy has been shown to reduce cardiovascular adverse events in PAD patients and is, therefore, the current gold standard, other drugs have also been investigated. The COMPASS and VOGAYER-PAD trials studied direct oral anticoagulation plus aspirin vs. aspirin alone and showed lower adverse limb and cardiovascular events for the first group [27,28]. However, bleeding complications were higher in the group using anticoagulation. Therefore, a dedicated randomized trial to clarify the effects and risks of SAPT and DAPT after endovascular treatment, with enough power to include subgroup analyses, remains essential.

This study has several limitations. First, owing to the retrospective nature of the study, DAPT durations varied. The number of patients with 12-month DAPT was small. This, however, reflects daily practice and emphasizes the lack of corresponding protocols.

Second, tracing medication use up to 1 year of follow-up was not always possible, which resulted in shorter follow-up for these patients.

Third, subgroup analyses of patients with BTK treatment were performed, but the number of patients, especially in the DAPT group, was quite small to draw firm conclusions.

Fourth, nonresponsiveness to acetylsalicylic acid or clopidogrel was not tested.

Fifth, prescribing patterns varied between the included centers. However, this was taken into account in the multivariate analyses.

Finally, MALE included thromboembolic complications of the target vessel, but no proximal or distal embolic debris.

## 5. Conclusions

No differences were observed between SAPT and DAPT on 1-, 3-, and 12-month MALE, MACE, and bleeding complications after infrainguinal endovascular revascularizations in this multicenter retrospective study. Subgroup analysis of patients who underwent femoropopliteal treatment also showed no differences, not even when corrected for stent use, stent type, and stent length. On the other hand, subgroup analysis of patients with BTK treatment suggests that DAPT is favorable over SAPT regarding 12-month MALE rate, even though subgroups were small. More robust data are, however, needed to further clarify the eventual added value of DAPT after infrainguinal endovascular treatment.

## Figures and Tables

**Table 1:** Definitions of the end points.

Major Adverse Limb Events	Major Amputation (Above the Ankle), Target Lesion Revascularization, or Target Vessel Revascularization (Endovascular or Surgical)
Major Adverse Cardiovascular Events	All-cause death, myocardial infarction, or stroke
Myocardial infarction	Based on clinical symptoms, electrocardiogram, and laboratory findings
Stroke	Transient ischemic attack or cerebrovascular accident, based on symptoms and radiologic findings
Bleeding Academic Research Consortium (BARC) [14]	Not actionable Any overt, actionable sign of hemorrhage requiring nonsurgical, medical intervention, or leading to hospitalization or increased level of care or prompting evaluation Overt bleeding plus hemoglobin drop >3 g/dL, any transfusion, bleeding requiring surgical intervention or intravenous vasoactive agents, intracranial hemorrhage, subcategories confirmed by autopsy, imaging, or lumbar puncture, intraocular bleed comprising vision Not applicable Fatal bleeding

**Table 2:** Baseline characteristics per patient.

Characteristic	SAPT (*n* = 149)	DAPT (*n* = 88)	*p*-Value
Age, mean ± SD (years)	74.4 ± 11.1	68.7 ± 12.1	<0.01
Male sex	87 (58)	49 (56)	0.68
Tobacco use (former/current)	99 (66)	75 (85)	<0.01
Diabetes mellitus	68 (46)	40 (46)	0.98
Hyperlipidemia	65 (44)	61 (69)	<0.01
Hypertension	98 (66)	58 (66)	0.98
Ischemic heart disease	52 (35)	33 (38)	0.69
Prior stroke	30 (20)	19 (22)	0.79
Renal failure, eGFR < 30 mL/min/1.73 m^2^	14 (9)	9 (10)	0.84
Dialysis dependent renal failure	5 (3)	3 (3)	1
ASA class			0.25
1	7 (5)	0 (0)	
2	60 (40)	37 (42)	
3	78 (52)	47 (53)	
4	4 (3)	4 (5)	
Rutherford category			0.24
1	2 (1)	1 (1)	
2	3 (2)	1 (1)	
3	38 (26)	22 (25)	
4	14 (9)	21 (24)	
5	74 (50)	38 (43)	
6	18 (12)	5 (6)	
Type of treatment			0.59
Fempop only	86 (57)	59 (67)	
Fempop/BTK	36 (24)	24 (27)	
BTK only	27 (18)	5 (6)	

ASA = American Society of Anesthesiologists Classification; BTK = below-the-knee; DAPT = dual antiplatelet therapy; eGFR = estimated glomerular filtration rate; fempop = femoropopliteal including superficial femoral artery, popliteal artery segment 1 and 2; SAPT = single antiplatelet therapy; SD = standard deviation.

**Table 3:** Characteristics of the 258 treated limbs.

Characteristic	SAPT (*n* = 159)	DAPT (*n* = 99)	*p*-Value
Rutherford category			0.28
1	2 (1)	1 (1)	
2	3 (2)	1 (1)	
3	44 (28)	26 (26)	
4	15 (9)	23 (23)	
5	76 (48)	43 (43)	
6	19 (12)	5 (5)	
Type of treatment			0.85
Fempop only	94 (59)	64 (65)	
Fempop/BTK	38 (24)	27 (27)	
BTK only	27 (17)	8 (8)	
Stent use fempop (fempop only and fempop/BTK treated limbs)	*n* = 132	*n* = 91	<0.01
	53 (40)	69 (76)	
Stent type fempop	*n* = 53	*n* = 68	
BMS	39 (74)	31 (46)	
DES	11 (21)	30 (44)	
BMS/DES combination	3 (6)	7 (10)	
Stent length fempop in cm (mean ± SD) (fempop and fempop/BTK treated limbs)	*n* = 52 *	*n* = 65 ^¥^,*	0.65
	15.7 ± 9.2	16.5 ± 9.2	

BMS = bare metal stent; BTK = below-the-knee, peroneal artery; DAPT = dual antiplatelet therapy; DES = drug-eluting stent; fempop = femoropopliteal including superficial femoral artery, popliteal artery segment 1 and 2; *n* = number; SAPT = single antiplatelet therapy; SD = standard deviation. ^¥^ Data on stent type was missing for 1 patient in the DAPT group. * Data on stent length was missing for 1 patient in the SAPT and 4 patients in the DAPT group.

**Table 4:** MALE outcomes at 1, 3, and 12 months between SAPT (*n* = 159) and DAPT (*n* = 99).

Outcome	Univariate		Multivariate	
	HR (95% CI)	*p*-Value	HR (95% CI)	*p*-Value
*1-month MALE*				
Overall cohort	0.37 (0.10–1.28)	0.12	0.35 (0.10–1.23)	0.1
Subgroup fempop	0.43 (0.12–1.58)	0.2	0.39 (0.11–1.42)	0.15
(SAPT *n* = 132,				
DAPT *n* = 91)				
Subgroup BTK	0.02 (0.00–9.60)	0.22	0.02 (0.00–9.60)	0.22
(SAPT *n* = 65,				
DAPT *n* = 35)				
*1-month major amputation*	0.26 (0.03–2.18)	0.22	0.26 (0.03–2.19)	0.22
*1-month TLR/TVR*	0.40 (0.09–1.89)	0.25	0.40 (0.09–1.89)	0.25
*3-month MALE*				
Overall cohort	0.47 (0.19–1.16)	0.1	0.45 (0.18–1.12)	0.09
Subgroup fempop	0.567 (0.220–1.462)	0.24	0.594 (0.230–1.534)	0.28
(SAPT *n* = 132,				
DAPT *n* = 91)				
Subgroup BTK	0.30 (0.07–1.35)	0.12	0.29 (0.06–1.29)	0.1
(SAPT *n* = 65,				
DAPT *n* = 35)				
*3-month major amputation*	0.90 (0.26–3.06)	0.86	0.90 (0.26–3.07	0.87
*3-month TLR/TVR*	0.31 (0.09–1.08)	0.07	0.31 (0.09–1.08)	0.07
*12-month MALE*				
Overall cohort	0.72 (0.40–1.29)	0.27	0.70 (0.39–1.25)	0.23
Subgroup fempop	0.81 (0.43–1.51)	0.5	0.76 (0.40–1.42)	0.38
(SAPT *n* = 132,				
DAPT *n* = 91)				
Subgroup BTK	0.45 (0.17–1.21)	0.12	0.33 (0.12-0.95)	0.04
(SAPT *n* = 65,				
DAPT *n* = 35)				
*12-month major amputation*	1.07 (0.42–2.77)	0.88	1.14 (0.44–2.95)	0.8
*12-month TLR/TVR*	0.61 (0.31–1.21)	0.16	0.61 (0.31–1.21)	0.16

BTK = below-the-knee; CI = confidence interval; DAPT = dual antiplatelet therapy; fempop = femoropopliteal including superficial femoral artery, popliteal artery segment 1 and 2; HR = hazard ratio; MALE = major adverse limb event; *n* = number; SAPT = single antiplatelet therapy; TLR = target lesion revascularization; TVR = target vessel revascularization.

**Table 5:** MACE outcomes at 1, 3, and 12 months between SAPT (*n* = 149) and DAPT (*n* = 88).

Outcome	Univariate		Multivariate	
	HR (95% CI)	*p*-Value	HR (95% CI)	*p*-Value
*1-month*				
MACE	1.12 (0.19–6.71)	0.90	1.12 (0.19–6.71)	0.90
Death	1.68 (0.11–26.80)	0.72	1.68 (0.11–26.80)	0.72
*3-month*				
MACE	0.63 (0.17–2.36)	0.49	0.64 (0.17–2.41)	0.51
Death	0.56 (0.11–2.75)	0.47	0.56 (0.11–2.78)	0.48
Myocardial infarction	1.67 (0.10–26.64)	0.72	1.67 (0.10–26.638)	0.72
Stroke	0.02 (0.00–46,5348)	0.66	0.02 (0.00–46,5348)	0.66
*12-month*				
MACE	1.41 (0.69–2.88)	0.35	1.50 (0.73–3.08)	0.27
Death	1.12 (0.47–2.71)	0.80	1.01 (0.42–2.44)	0.99
Myocardial infarction	3.38 (0.81–14.18)	0.10	3.80 (0.90–16.07)	0.07
Stroke	1.57 (0.35–7.04)	0.56	1.74 (0.38–7.89)	0.47

CI = confidence interval; DAPT = dual antiplatelet therapy; HR = hazard ratio; MACE = major adverse cardiovascular event; SAPT = single antiplatelet therapy.
